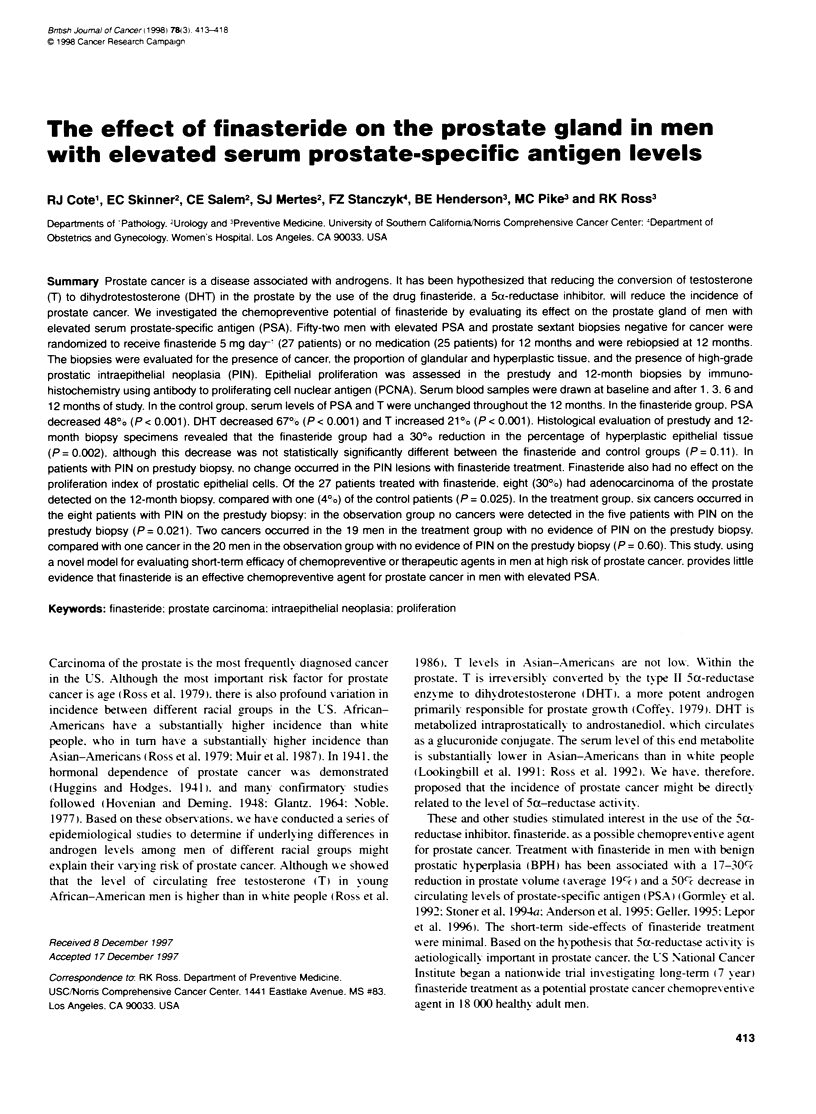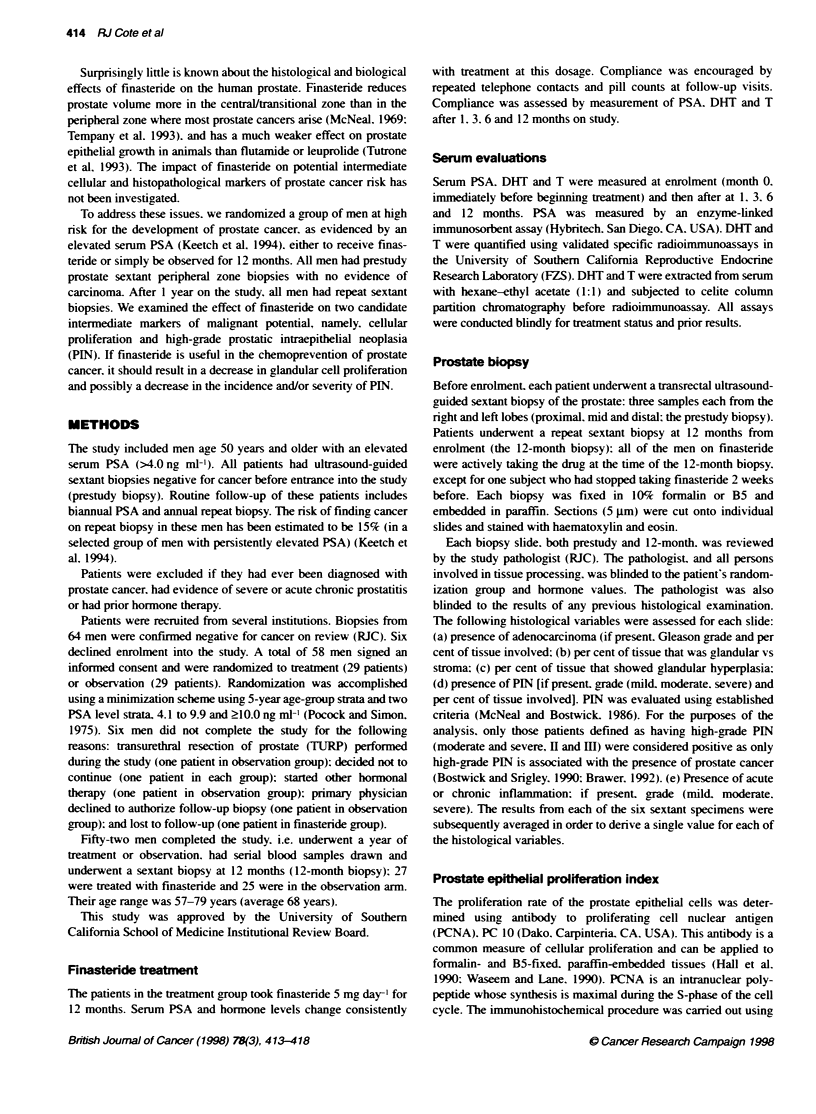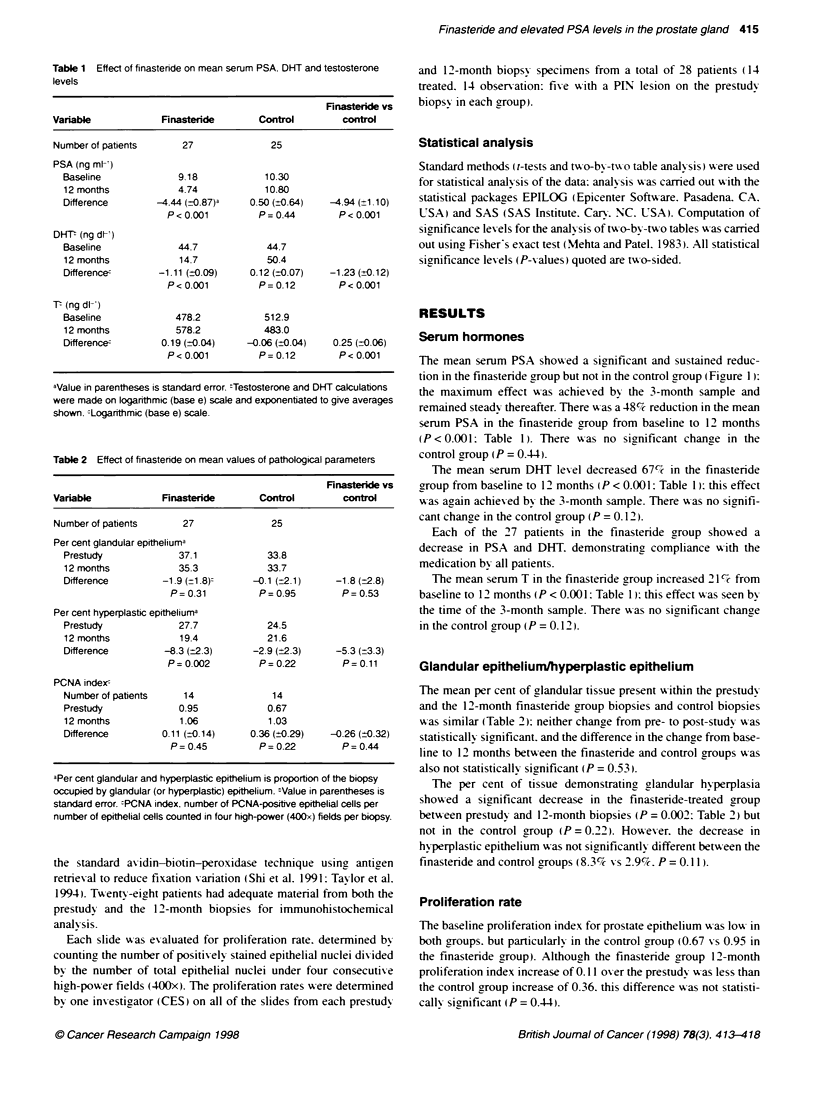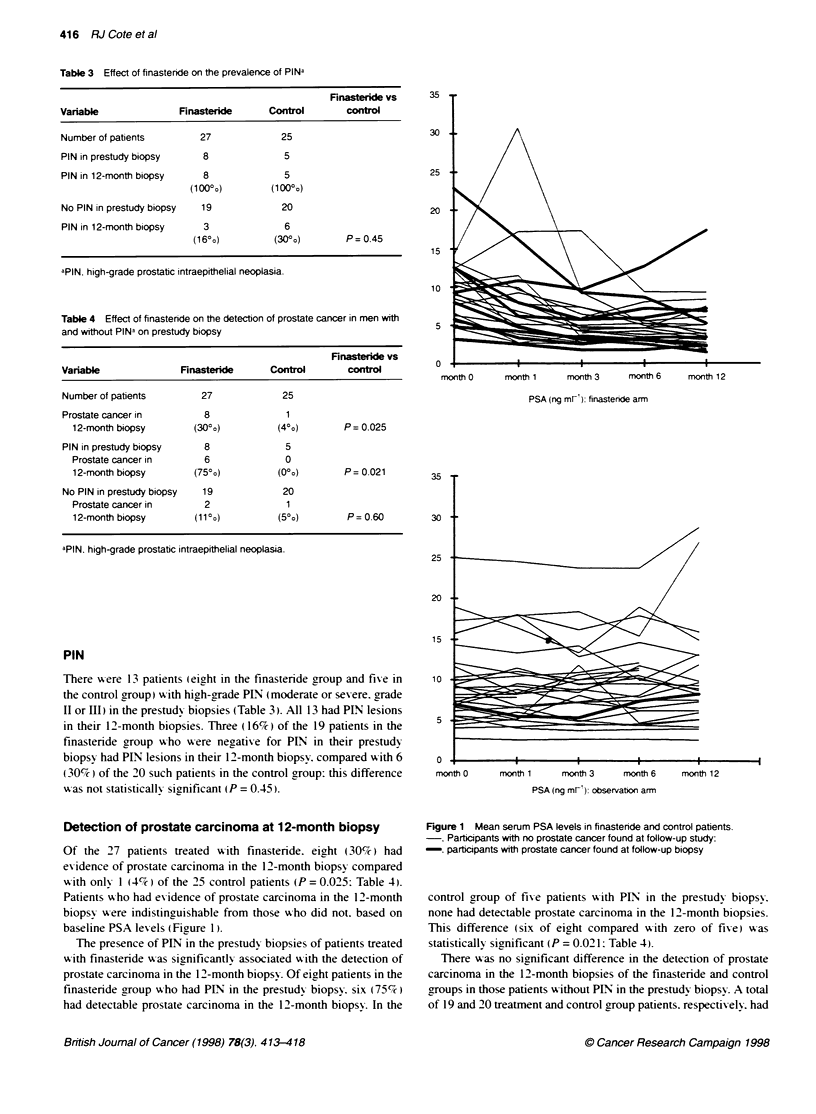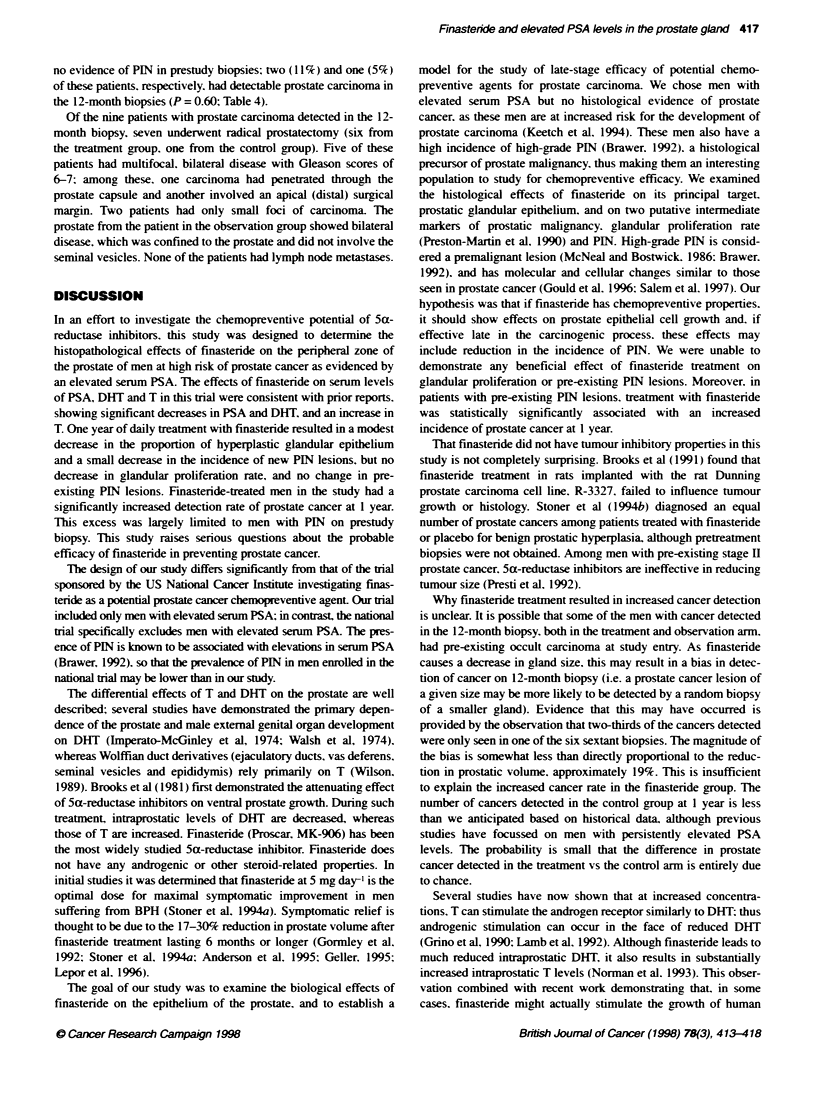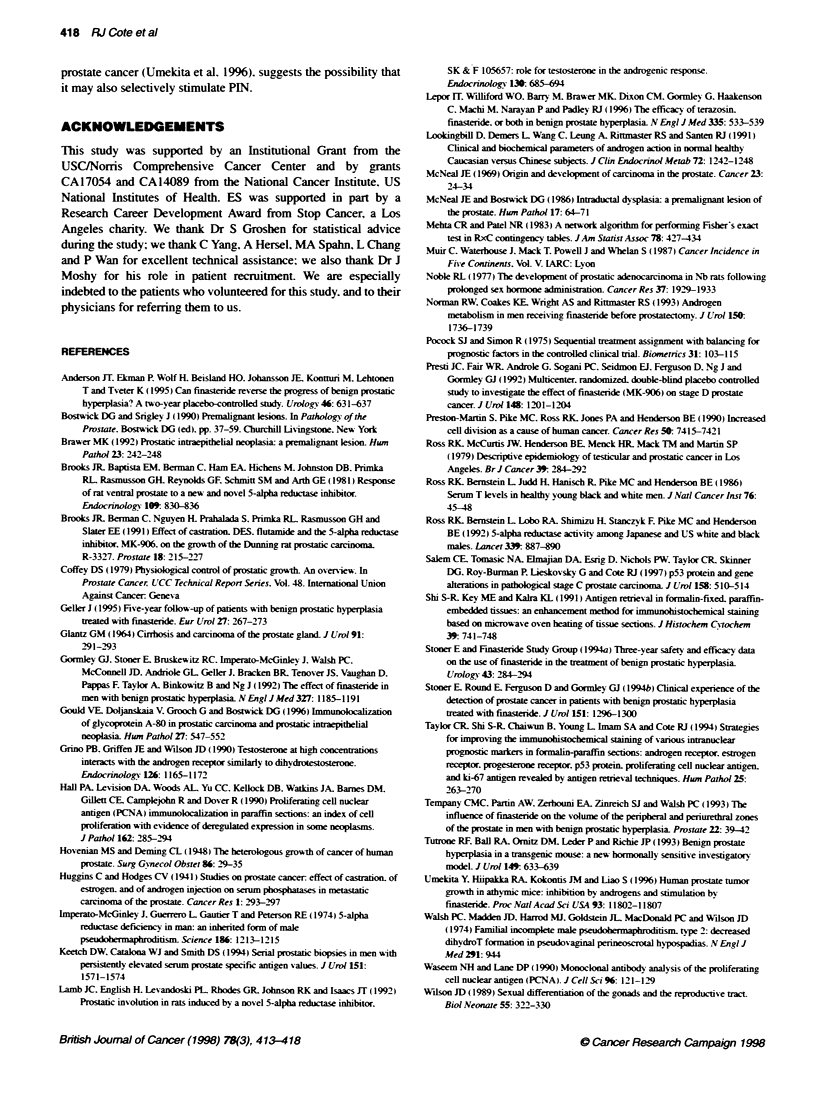# The effect of finasteride on the prostate gland in men with elevated serum prostate-specific antigen levels.

**DOI:** 10.1038/bjc.1998.508

**Published:** 1998-08

**Authors:** R. J. Cote, E. C. Skinner, C. E. Salem, S. J. Mertes, F. Z. Stanczyk, B. E. Henderson, M. C. Pike, R. K. Ross

**Affiliations:** Department of Pathology, University of Southern California/Norris Comprehensive Cancer Center, Los Angeles 90033, USA.

## Abstract

Prostate cancer is a disease associated with androgens. It has been hypothesized that reducing the conversion of testosterone (T) to dihydrotestosterone (DHT) in the prostate by the use of the drug finasteride, a 5alpha-reductase inhibitor, will reduce the incidence of prostate cancer. We investigated the chemopreventive potential of finasteride by evaluating its effect on the prostate gland of men with elevated serum prostate-specific antigen (PSA). Fifty-two men with elevated PSA and prostate sextant biopsies negative for cancer were randomized to receive finasteride 5 mg day(-1) (27 patients) or no medication (25 patients) for 12 months and were rebiopsied at 12 months. The biopsies were evaluated for the presence of cancer, the proportion of glandular and hyperplastic tissue, and the presence of high-grade prostatic intraepithelial neoplasia (PIN). Epithelial proliferation was assessed in the prestudy and 12-month biopsies by immunohistochemistry using antibody to proliferating cell nuclear antigen (PCNA). Serum blood samples were drawn at baseline and after 1, 3, 6 and 12 months of study. In the control group, serum levels of PSA and T were unchanged throughout the 12 months. In the finasteride group, PSA decreased 48% (P < 0.001), DHT decreased 67% (P < 0.001) and T increased 21% (P < 0.001). Histological evaluation of prestudy and 12-month biopsy specimens revealed that the finasteride group had a 30% reduction in the percentage of hyperplastic epithelial tissue (P = 0.002), although this decrease was not statistically significantly different between the finasteride and control groups (P = 0.11). In patients with PIN on prestudy biopsy, no change occurred in the PIN lesions with finasteride treatment. Finasteride also had no effect on the proliferation index of prostatic epithelial cells. Of the 27 patients treated with finasteride, eight (30%) had adenocarcinoma of the prostate detected on the 12-month biopsy, compared with one (4%) of the control patients (P = 0.025). In the treatment group, six cancers occurred in the eight patients with PIN on the prestudy biopsy; in the observation group no cancers were detected in the five patients with PIN on the prestudy biopsy (P = 0.021). Two cancers occurred in the 19 men in the treatment group with no evidence of PIN on the prestudy biopsy, compared with one cancer in the 20 men in the observation group with no evidence of PIN on the prestudy biopsy (P = 0.60). This study, using a novel model for evaluating short-term efficacy of chemopreventive or therapeutic agents in men at high risk of prostate cancer, provides little evidence that finasteride is an effective chemopreventive agent for prostate cancer in men with elevated PSA.


British Journal of Cancer (1998) 78(3). 413–418
© 1998 Cancer Research Campaign

### The effect of finasteride on the prostate gland in men with elevated serum prostate-specific antigen levels

**RJ Cote[1], EC Skinner[2], CE Salem[2], SJ Mertes[2], FZ Stanczyk[4], BE Henderson[3], MC Pike[3] and RK Ross[3]**

Departments of [1]Pathology. [2]Urology and [3]Preventive Medicine. University of Southern California/Norris Comprehensive Cancer Center: [4]Department of Obstetrics and Gynecology. Women's Hospital. Los Angeles. CA 90033. USA

**Summary** Prostate cancer is a disease associated with androgens. It has been hypothesized that reducing the conversion of testosterone (T) to dihydrotestosterone (DHT) in the prostate by the use of the drug finasteride, a 5α-reductase inhibitor, will reduce the incidence of prostate cancer. We investigated the chemopreventive potential of finasteride by evaluating its effect on the prostate gland of men with elevated serum prostate-specific antigen (PSA). Fifty-two men with elevated PSA and prostate sextant biopsies negative for cancer were randomized to receive finasteride 5 mg day$^{-1}$ (27 patients) or no medication (25 patients) for 12 months and were rebiopsied at 12 months. The biopsies were evaluated for the presence of cancer, the proportion of glandular and hyperplastic tissue, and the presence of high-grade prostatic intraepithelial neoplasia (PIN). Epithelial proliferation was assessed in the prestudy and 12-month biopsies by immunohistochemistry using antibody to proliferating cell nuclear antigen (PCNA). Serum blood samples were drawn at baseline and after 1, 3, 6 and 12 months of study. In the control group, serum levels of PSA and T were unchanged throughout the 12 months. In the finasteride group, PSA decreased 48% ($P < 0.001$). DHT decreased 67% ($P < 0.001$) and T increased 21% ($P < 0.001$). Histological evaluation of prestudy and 12-month biopsy specimens revealed that the finasteride group had a 30% reduction in the percentage of hyperplastic epithelial tissue ($P = 0.002$), although this decrease was not statistically significantly different between the finasteride and control groups ($P = 0.11$). In patients with PIN on prestudy biopsy, no change occurred in the PIN lesions with finasteride treatment. Finasteride also had no effect on the proliferation index of prostatic epithelial cells. Of the 27 patients treated with finasteride, eight (30%) had adenocarcinoma of the prostate detected on the 12-month biopsy, compared with one (4%) of the control patients ($P = 0.025$). In the treatment group, six cancers occurred in the eight patients with PIN on the prestudy biopsy; in the observation group no cancers were detected in the five patients with PIN on the prestudy biopsy ($P = 0.021$). Two cancers occurred in the 19 men in the treatment group with no evidence of PIN on the prestudy biopsy, compared with one cancer in the 20 men in the observation group with no evidence of PIN on the prestudy biopsy ($P = 0.60$). This study, using a novel model for evaluating short-term efficacy of chemopreventive or therapeutic agents in men at high risk of prostate cancer, provides little evidence that finasteride is an effective chemopreventive agent for prostate cancer in men with elevated PSA.

**Keywords:** finasteride; prostate carcinoma; intraepithelial neoplasia; proliferation

Carcinoma of the prostate is the most frequently diagnosed cancer in the US. Although the most important risk factor for prostate cancer is age (Ross et al. 1979), there is also profound variation in incidence between different racial groups in the US. African–Americans have a substantially higher incidence than white people, who in turn have a substantially higher incidence than Asian–Americans (Ross et al. 1979; Muir et al. 1987). In 1941, the hormonal dependence of prostate cancer was demonstrated (Huggins and Hodges, 1941), and many confirmatory studies followed (Hovenian and Deming, 1948; Glantz, 1964; Noble, 1977). Based on these observations, we have conducted a series of epidemiological studies to determine if underlying differences in androgen levels among men of different racial groups might explain their varying risk of prostate cancer. Although we showed that the level of circulating free testosterone (T) in young African–American men is higher than in white people (Ross et al. 1986), T levels in Asian–Americans are not low. Within the prostate, T is irreversibly converted by the type II 5α-reductase enzyme to dihydrotestosterone (DHT), a more potent androgen primarily responsible for prostate growth (Coffey, 1979). DHT is metabolized intraprostatically to androstanediol, which circulates as a glucuronide conjugate. The serum level of this end metabolite is substantially lower in Asian–Americans than in white people (Lookingbill et al. 1991; Ross et al. 1992). We have, therefore, proposed that the incidence of prostate cancer might be directly related to the level of 5α–reductase activity.

*Received 8 December 1997*
*Accepted 17 December 1997*

*Correspondence to:* RK Ross, Department of Preventive Medicine, USC/Norris Comprehensive Cancer Center, 1441 Eastlake Avenue, MS #83, Los Angeles, CA 90033, USA

These and other studies stimulated interest in the use of the 5α-reductase inhibitor, finasteride, as a possible chemopreventive agent for prostate cancer. Treatment with finasteride in men with benign prostatic hyperplasia (BPH) has been associated with a 17–30% reduction in prostate volume (average 19%) and a 50% decrease in circulating levels of prostate-specific antigen (PSA) (Gormley et al. 1992; Stoner et al. 1994*a*; Anderson et al. 1995; Geller, 1995; Lepor et al. 1996). The short-term side-effects of finasteride treatment were minimal. Based on the hypothesis that 5α-reductase activity is aetiologically important in prostate cancer, the US National Cancer Institute began a nationwide trial investigating long-term (7 year) finasteride treatment as a potential prostate cancer chemopreventive agent in 18 000 healthy adult men.

Surprisingly little is known about the histological and biological effects of finasteride on the human prostate. Finasteride reduces prostate volume more in the central/transitional zone than in the peripheral zone where most prostate cancers arise (McNeal, 1969; Tempany et al, 1993), and has a much weaker effect on prostate epithelial growth in animals than flutamide or leuprolide (Tutrone et al, 1993). The impact of finasteride on potential intermediate cellular and histopathological markers of prostate cancer risk has not been investigated.

To address these issues, we randomized a group of men at high risk for the development of prostate cancer, as evidenced by an elevated serum PSA (Keetch et al, 1994), either to receive finasteride or simply be observed for 12 months. All men had prestudy prostate sextant peripheral zone biopsies with no evidence of carcinoma. After 1 year on the study, all men had repeat sextant biopsies. We examined the effect of finasteride on two candidate intermediate markers of malignant potential, namely, cellular proliferation and high-grade prostatic intraepithelial neoplasia (PIN). If finasteride is useful in the chemoprevention of prostate cancer, it should result in a decrease in glandular cell proliferation and possibly a decrease in the incidence and/or severity of PIN.

### METHODS

The study included men age 50 years and older with an elevated serum PSA (>4.0 ng ml$^{-1}$). All patients had ultrasound-guided sextant biopsies negative for cancer before entrance into the study (prestudy biopsy). Routine follow-up of these patients includes biannual PSA and annual repeat biopsy. The risk of finding cancer on repeat biopsy in these men has been estimated to be 15% (in a selected group of men with persistently elevated PSA) (Keetch et al, 1994).

Patients were excluded if they had ever been diagnosed with prostate cancer, had evidence of severe or acute chronic prostatitis or had prior hormone therapy.

Patients were recruited from several institutions. Biopsies from 64 men were confirmed negative for cancer on review (RJC). Six declined enrolment into the study. A total of 58 men signed an informed consent and were randomized to treatment (29 patients) or observation (29 patients). Randomization was accomplished using a minimization scheme using 5-year age-group strata and two PSA level strata, 4.1 to 9.9 and ≥10.0 ng ml$^{-1}$ (Pocock and Simon, 1975). Six men did not complete the study for the following reasons: transurethral resection of prostate (TURP) performed during the study (one patient in observation group); decided not to continue (one patient in each group); started other hormonal therapy (one patient in observation group); primary physician declined to authorize follow-up biopsy (one patient in observation group); and lost to follow-up (one patient in finasteride group).

Fifty-two men completed the study, i.e. underwent a year of treatment or observation, had serial blood samples drawn and underwent a sextant biopsy at 12 months (12-month biopsy); 27 were treated with finasteride and 25 were in the observation arm. Their age range was 57–79 years (average 68 years).

This study was approved by the University of Southern California School of Medicine Institutional Review Board.

### Finasteride treatment

The patients in the treatment group took finasteride 5 mg day$^{-1}$ for 12 months. Serum PSA and hormone levels change consistently with treatment at this dosage. Compliance was encouraged by repeated telephone contacts and pill counts at follow-up visits. Compliance was assessed by measurement of PSA, DHT and T after 1, 3, 6 and 12 months on study.

### Serum evaluations

Serum PSA, DHT and T were measured at enrolment (month 0, immediately before beginning treatment) and then after at 1, 3, 6 and 12 months. PSA was measured by an enzyme-linked immunosorbent assay (Hybritech, San Diego, CA, USA). DHT and T were quantified using validated specific radioimmunoassays in the University of Southern California Reproductive Endocrine Research Laboratory (FZS). DHT and T were extracted from serum with hexane–ethyl acetate (1:1) and subjected to celite column partition chromatography before radioimmunoassay. All assays were conducted blindly for treatment status and prior results.

### Prostate biopsy

Before enrolment, each patient underwent a transrectal ultrasound-guided sextant biopsy of the prostate: three samples each from the right and left lobes (proximal, mid and distal; the prestudy biopsy). Patients underwent a repeat sextant biopsy at 12 months from enrolment (the 12-month biopsy); all of the men on finasteride were actively taking the drug at the time of the 12-month biopsy, except for one subject who had stopped taking finasteride 2 weeks before. Each biopsy was fixed in 10% formalin or B5 and embedded in paraffin. Sections (5 μm) were cut onto individual slides and stained with haematoxylin and eosin.

Each biopsy slide, both prestudy and 12-month, was reviewed by the study pathologist (RJC). The pathologist, and all persons involved in tissue processing, was blinded to the patient's randomization group and hormone values. The pathologist was also blinded to the results of any previous histological examination. The following histological variables were assessed for each slide: (a) presence of adenocarcinoma (if present, Gleason grade and per cent of tissue involved; (b) per cent of tissue that was glandular vs stroma; (c) per cent of tissue that showed glandular hyperplasia; (d) presence of PIN [if present, grade (mild, moderate, severe) and per cent of tissue involved]. PIN was evaluated using established criteria (McNeal and Bostwick, 1986). For the purposes of the analysis, only those patients defined as having high-grade PIN (moderate and severe, II and III) were considered positive as only high-grade PIN is associated with the presence of prostate cancer (Bostwick and Srigley, 1990; Brawer, 1992). (e) Presence of acute or chronic inflammation; if present, grade (mild, moderate, severe). The results from each of the six sextant specimens were subsequently averaged in order to derive a single value for each of the histological variables.

### Prostate epithelial proliferation index

The proliferation rate of the prostate epithelial cells was determined using antibody to proliferating cell nuclear antigen (PCNA), PC 10 (Dako, Carpinteria, CA, USA). This antibody is a common measure of cellular proliferation and can be applied to formalin- and B5-fixed, paraffin-embedded tissues (Hall et al, 1990; Waseem and Lane, 1990). PCNA is an intranuclear polypeptide whose synthesis is maximal during the S-phase of the cell cycle. The immunohistochemical procedure was carried out using

 © Cancer Research Campaign 1998

**Table 1** Effect of finasteride on mean serum PSA, DHT and testosterone levels

| Variable | Finasteride | Control | Finasteride vs control |
|---|---|---|---|
| Number of patients | 27 | 25 | |
| PSA (ng ml⁻¹) | | | |
| Baseline | 9.18 | 10.30 | |
| 12 months | 4.74 | 10.80 | |
| Difference | –4.44 (±0.87)[a] | 0.50 (±0.64) | –4.94 (±1.10) |
| | $P < 0.001$ | $P = 0.44$ | $P < 0.001$ |
| DHT[b] (ng dl⁻¹) | | | |
| Baseline | 44.7 | 44.7 | |
| 12 months | 14.7 | 50.4 | |
| Difference[c] | –1.11 (±0.09) | 0.12 (±0.07) | –1.23 (±0.12) |
| | $P < 0.001$ | $P = 0.12$ | $P < 0.001$ |
| T[b] (ng dl⁻¹) | | | |
| Baseline | 478.2 | 512.9 | |
| 12 months | 578.2 | 483.0 | |
| Difference[c] | 0.19 (±0.04) | –0.06 (±0.04) | 0.25 (±0.06) |
| | $P < 0.001$ | $P = 0.12$ | $P < 0.001$ |

[a]Value in parentheses is standard error. [b]Testosterone and DHT calculations were made on logarithmic (base e) scale and exponentiated to give averages shown. [c]Logarithmic (base e) scale.

**Table 2** Effect of finasteride on mean values of pathological parameters

| Variable | Finasteride | Control | Finasteride vs control |
|---|---|---|---|
| Number of patients | 27 | 25 | |
| Per cent glandular epithelium[a] | | | |
| Prestudy | 37.1 | 33.8 | |
| 12 months | 35.3 | 33.7 | |
| Difference | –1.9 (±1.8)[b] | –0.1 (±2.1) | –1.8 (±2.8) |
| | $P = 0.31$ | $P = 0.95$ | $P = 0.53$ |
| Per cent hyperplastic epithelium[a] | | | |
| Prestudy | 27.7 | 24.5 | |
| 12 months | 19.4 | 21.6 | |
| Difference | –8.3 (±2.3) | –2.9 (±2.3) | –5.3 (±3.3) |
| | $P = 0.002$ | $P = 0.22$ | $P = 0.11$ |
| PCNA index[c] | | | |
| Number of patients | 14 | 14 | |
| Prestudy | 0.95 | 0.67 | |
| 12 months | 1.06 | 1.03 | |
| Difference | 0.11 (±0.14) | 0.36 (±0.29) | –0.26 (±0.32) |
| | $P = 0.45$ | $P = 0.22$ | $P = 0.44$ |

[a]Per cent glandular and hyperplastic epithelium is proportion of the biopsy occupied by glandular (or hyperplastic) epithelium. [b]Value in parentheses is standard error. [c]PCNA index, number of PCNA-positive epithelial cells per number of epithelial cells counted in four high-power (400×) fields per biopsy.

the standard avidin–biotin–peroxidase technique using antigen retrieval to reduce fixation variation (Shi et al, 1991; Taylor et al, 1994). Twenty-eight patients had adequate material from both the prestudy and the 12-month biopsies for immunohistochemical analysis.

Each slide was evaluated for proliferation rate, determined by counting the number of positively stained epithelial nuclei divided by the number of total epithelial nuclei under four consecutive high-power fields (400×). The proliferation rates were determined by one investigator (CES) on all of the slides from each prestudy and 12-month biopsy specimens from a total of 28 patients (14 treated, 14 observation; five with a PIN lesion on the prestudy biopsy in each group).

### Statistical analysis

Standard methods (*t*-tests and two-by-two table analysis) were used for statistical analysis of the data; analysis was carried out with the statistical packages EPILOG (Epicenter Software, Pasadena, CA, USA) and SAS (SAS Institute, Cary, NC, USA). Computation of significance levels for the analysis of two-by-two tables was carried out using Fisher's exact test (Mehta and Patel, 1983). All statistical significance levels (*P*-values) quoted are two-sided.

### RESULTS

### Serum hormones

The mean serum PSA showed a significant and sustained reduction in the finasteride group but not in the control group (Figure 1); the maximum effect was achieved by the 3-month sample and remained steady thereafter. There was a 48% reduction in the mean serum PSA in the finasteride group from baseline to 12 months ($P < 0.001$; Table 1). There was no significant change in the control group ($P = 0.44$).

The mean serum DHT level decreased 67% in the finasteride group from baseline to 12 months ($P < 0.001$; Table 1); this effect was again achieved by the 3-month sample. There was no significant change in the control group ($P = 0.12$).

Each of the 27 patients in the finasteride group showed a decrease in PSA and DHT, demonstrating compliance with the medication by all patients.

The mean serum T in the finasteride group increased 21% from baseline to 12 months ($P < 0.001$; Table 1); this effect was seen by the time of the 3-month sample. There was no significant change in the control group ($P = 0.12$).

### Glandular epithelium/hyperplastic epithelium

The mean per cent of glandular tissue present within the prestudy and the 12-month finasteride group biopsies and control biopsies was similar (Table 2); neither change from pre- to post-study was statistically significant, and the difference in the change from baseline to 12 months between the finasteride and control groups was also not statistically significant ($P = 0.53$).

The per cent of tissue demonstrating glandular hyperplasia showed a significant decrease in the finasteride-treated group between prestudy and 12-month biopsies ($P = 0.002$; Table 2) but not in the control group ($P = 0.22$). However, the decrease in hyperplastic epithelium was not significantly different between the finasteride and control groups (8.3% vs 2.9%, $P = 0.11$).

### Proliferation rate

The baseline proliferation index for prostate epithelium was low in both groups, but particularly in the control group (0.67 vs 0.95 in the finasteride group). Although the finasteride group 12-month proliferation index increase of 0.11 over the prestudy was less than the control group increase of 0.36, this difference was not statistically significant ($P = 0.44$).

**Table 3** Effect of finasteride on the prevalence of PIN[a]

| Variable | Finasteride | Control | Finasteride vs control |
|---|---|---|---|
| Number of patients | 27 | 25 | |
| PIN in prestudy biopsy | 8 | 5 | |
| PIN in 12-month biopsy | 8 (100%) | 5 (100%) | |
| No PIN in prestudy biopsy | 19 | 20 | |
| PIN in 12-month biopsy | 3 (16%) | 6 (30%) | $P = 0.45$ |

[a]PIN, high-grade prostatic intraepithelial neoplasia.

**Table 4** Effect of finasteride on the detection of prostate cancer in men with and without PIN[a] on prestudy biopsy

| Variable | Finasteride | Control | Finasteride vs control |
|---|---|---|---|
| Number of patients | 27 | 25 | |
| Prostate cancer in 12-month biopsy | 8 (30%) | 1 (4%) | $P = 0.025$ |
| PIN in prestudy biopsy | 8 | 5 | |
| Prostate cancer in 12-month biopsy | 6 (75%) | 0 (0%) | $P = 0.021$ |
| No PIN in prestudy biopsy | 19 | 20 | |
| Prostate cancer in 12-month biopsy | 2 (11%) | 1 (5%) | $P = 0.60$ |

[a]PIN, high-grade prostatic intraepithelial neoplasia.

### PIN

There were 13 patients (eight in the finasteride group and five in the control group) with high-grade PIN (moderate or severe, grade II or III) in the prestudy biopsies (Table 3). All 13 had PIN lesions in their 12-month biopsies. Three (16%) of the 19 patients in the finasteride group who were negative for PIN in their prestudy biopsy had PIN lesions in their 12-month biopsy, compared with 6 (30%) of the 20 such patients in the control group; this difference was not statistically significant ($P = 0.45$).

### Detection of prostate carcinoma at 12-month biopsy

Of the 27 patients treated with finasteride, eight (30%) had evidence of prostate carcinoma in the 12-month biopsy compared with only 1 (4%) of the 25 control patients ($P = 0.025$; Table 4). Patients who had evidence of prostate carcinoma in the 12-month biopsy were indistinguishable from those who did not, based on baseline PSA levels (Figure 1).

The presence of PIN in the prestudy biopsies of patients treated with finasteride was significantly associated with the detection of prostate carcinoma in the 12-month biopsy. Of eight patients in the finasteride group who had PIN in the prestudy biopsy, six (75%) had detectable prostate carcinoma in the 12-month biopsy. In the

**Figure 1** Mean serum PSA levels in finasteride and control patients. —, Participants with no prostate cancer found at follow-up study; ▬, participants with prostate cancer found at follow-up biopsy control group of five patients with PIN in the prestudy biopsy, none had detectable prostate carcinoma in the 12-month biopsies. This difference (six of eight compared with zero of five) was statistically significant ($P = 0.021$; Table 4).

There was no significant difference in the detection of prostate carcinoma in the 12-month biopsies of the finasteride and control groups in those patients without PIN in the prestudy biopsy. A total of 19 and 20 treatment and control group patients, respectively, had

© Cancer Research Campaign 1998

no evidence of PIN in prestudy biopsies; two (11%) and one (5%) of these patients, respectively, had detectable prostate carcinoma in the 12-month biopsies ($P = 0.60$; Table 4).

Of the nine patients with prostate carcinoma detected in the 12-month biopsy, seven underwent radical prostatectomy (six from the treatment group, one from the control group). Five of these patients had multifocal, bilateral disease with Gleason scores of 6–7; among these, one carcinoma had penetrated through the prostate capsule and another involved an apical (distal) surgical margin. Two patients had only small foci of carcinoma. The prostate from the patient in the observation group showed bilateral disease, which was confined to the prostate and did not involve the seminal vesicles. None of the patients had lymph node metastases.

### DISCUSSION

In an effort to investigate the chemopreventive potential of 5α-reductase inhibitors, this study was designed to determine the histopathological effects of finasteride on the peripheral zone of the prostate of men at high risk of prostate cancer as evidenced by an elevated serum PSA. The effects of finasteride on serum levels of PSA, DHT and T in this trial were consistent with prior reports, showing significant decreases in PSA and DHT, and an increase in T. One year of daily treatment with finasteride resulted in a modest decrease in the proportion of hyperplastic glandular epithelium and a small decrease in the incidence of new PIN lesions, but no decrease in glandular proliferation rate, and no change in pre-existing PIN lesions. Finasteride-treated men in the study had a significantly increased detection rate of prostate cancer at 1 year. This excess was largely limited to men with PIN on prestudy biopsy. This study raises serious questions about the probable efficacy of finasteride in preventing prostate cancer.

The design of our study differs significantly from that of the trial sponsored by the US National Cancer Institute investigating finasteride as a potential prostate cancer chemopreventive agent. Our trial included only men with elevated serum PSA; in contrast, the national trial specifically excludes men with elevated serum PSA. The presence of PIN is known to be associated with elevations in serum PSA (Brawer, 1992), so that the prevalence of PIN in men enrolled in the national trial may be lower than in our study.

The differential effects of T and DHT on the prostate are well described; several studies have demonstrated the primary dependence of the prostate and male external genital organ development on DHT (Imperato-McGinley et al, 1974; Walsh et al, 1974), whereas Wolffian duct derivatives (ejaculatory ducts, vas deferens, seminal vesicles and epididymis) rely primarily on T (Wilson, 1989). Brooks et al (1981) first demonstrated the attenuating effect of 5α-reductase inhibitors on ventral prostate growth. During such treatment, intraprostatic levels of DHT are decreased, whereas those of T are increased. Finasteride (Proscar, MK-906) has been the most widely studied 5α-reductase inhibitor. Finasteride does not have any androgenic or other steroid-related properties. In initial studies it was determined that finasteride at 5 mg $day^{-1}$ is the optimal dose for maximal symptomatic improvement in men suffering from BPH (Stoner et al, 1994*a*). Symptomatic relief is thought to be due to the 17–30% reduction in prostate volume after finasteride treatment lasting 6 months or longer (Gormley et al, 1992; Stoner et al, 1994*a*; Anderson et al, 1995; Geller, 1995; Lepor et al, 1996).

The goal of our study was to examine the biological effects of finasteride on the epithelium of the prostate, and to establish a model for the study of late-stage efficacy of potential chemopreventive agents for prostate carcinoma. We chose men with elevated serum PSA but no histological evidence of prostate cancer, as these men are at increased risk for the development of prostate carcinoma (Keetch et al, 1994). These men also have a high incidence of high-grade PIN (Brawer, 1992), a histological precursor of prostate malignancy, thus making them an interesting population to study for chemopreventive efficacy. We examined the histological effects of finasteride on its principal target, prostatic glandular epithelium, and on two putative intermediate markers of prostatic malignancy, glandular proliferation rate (Preston-Martin et al, 1990) and PIN. High-grade PIN is considered a premalignant lesion (McNeal and Bostwick, 1986; Brawer, 1992), and has molecular and cellular changes similar to those seen in prostate cancer (Gould et al, 1996; Salem et al, 1997). Our hypothesis was that if finasteride has chemopreventive properties, it should show effects on prostate epithelial cell growth and, if effective late in the carcinogenic process, these effects may include reduction in the incidence of PIN. We were unable to demonstrate any beneficial effect of finasteride treatment on glandular proliferation or pre-existing PIN lesions. Moreover, in patients with pre-existing PIN lesions, treatment with finasteride was statistically significantly associated with an increased incidence of prostate cancer at 1 year.

That finasteride did not have tumour inhibitory properties in this study is not completely surprising. Brooks et al (1991) found that finasteride treatment in rats implanted with the rat Dunning prostate carcinoma cell line, R-3327, failed to influence tumour growth or histology. Stoner et al (1994*b*) diagnosed an equal number of prostate cancers among patients treated with finasteride or placebo for benign prostatic hyperplasia, although pretreatment biopsies were not obtained. Among men with pre-existing stage II prostate cancer, 5α-reductase inhibitors are ineffective in reducing tumour size (Presti et al, 1992).

Why finasteride treatment resulted in increased cancer detection is unclear. It is possible that some of the men with cancer detected in the 12-month biopsy, both in the treatment and observation arm, had pre-existing occult carcinoma at study entry. As finasteride causes a decrease in gland size, this may result in a bias in detection of cancer on 12-month biopsy (i.e. a prostate cancer lesion of a given size may be more likely to be detected by a random biopsy of a smaller gland). Evidence that this may have occurred is provided by the observation that two-thirds of the cancers detected were only seen in one of the six sextant biopsies. The magnitude of the bias is somewhat less than directly proportional to the reduction in prostatic volume, approximately 19%. This is insufficient to explain the increased cancer rate in the finasteride group. The number of cancers detected in the control group at 1 year is less than we anticipated based on historical data, although previous studies have focussed on men with persistently elevated PSA levels. The probability is small that the difference in prostate cancer detected in the treatment vs the control arm is entirely due to chance.

Several studies have now shown that at increased concentrations, T can stimulate the androgen receptor similarly to DHT; thus androgenic stimulation can occur in the face of reduced DHT (Grino et al, 1990; Lamb et al, 1992). Although finasteride leads to much reduced intraprostatic DHT, it also results in substantially increased intraprostatic T levels (Norman et al, 1993). This observation combined with recent work demonstrating that, in some cases, finasteride might actually stimulate the growth of human prostate cancer (Umekita et al. 1996), suggests the possibility that it may also selectively stimulate PIN.

### ACKNOWLEDGEMENTS

This study was supported by an Institutional Grant from the USC/Norris Comprehensive Cancer Center and by grants CA17054 and CA14089 from the National Cancer Institute, US National Institutes of Health. ES was supported in part by a Research Career Development Award from Stop Cancer, a Los Angeles charity. We thank Dr S Groshen for statistical advice during the study; we thank C Yang, A Hersel, MA Spahn, L Chang and P Wan for excellent technical assistance; we also thank Dr J Moshy for his role in patient recruitment. We are especially indebted to the patients who volunteered for this study, and to their physicians for referring them to us.

### REFERENCES

Anderson JT, Ekman P, Wolf H, Beisland HO, Johansson JE, Kontturi M, Lehtonen T and Tveter K (1995) Can finasteride reverse the progress of benign prostatic hyperplasia? A two-year placebo-controlled study. *Urology* **46**: 631–637

Bostwick DG and Srigley J (1990) Premalignant lesions. In *Pathology of the Prostate*, Bostwick DG (ed), pp. 37–59. Churchill Livingstone, New York

Brawer MK (1992) Prostatic intraepithelial neoplasia: a premalignant lesion. *Hum Pathol* **23**: 242–248

Brooks JR, Baptista EM, Berman C, Ham EA, Hichens M, Johnston DB, Primka RL, Rasmusson GH, Reynolds GF, Schmitt SM and Arth GE (1981) Response of rat ventral prostate to a new and novel 5-alpha reductase inhibitor. *Endocrinology* **109**: 830–836

Brooks JR, Berman C, Nguyen H, Prahalada S, Primka RL, Rasmusson GH and Slater EE (1991) Effect of castration, DES, flutamide and the 5-alpha reductase inhibitor, MK-906, on the growth of the Dunning rat prostatic carcinoma, R-3327. *Prostate* **18**: 215–227

Coffey DS (1979) Physiological control of prostatic growth. An overview. In *Prostate Cancer, UCC Technical Report Series*, Vol. 48. International Union Against Cancer: Geneva

Geller J (1995) Five-year follow-up of patients with benign prostatic hyperplasia treated with finasteride. *Eur Urol* **27**: 267–273

Glantz GM (1964) Cirrhosis and carcinoma of the prostate gland. *J Urol* **91**: 291–293

Gormley GJ, Stoner E, Bruskewitz RC, Imperato-McGinley J, Walsh PC, McConnell JD, Andriole GL, Geller J, Bracken BR, Tenover JS, Vaughan D, Pappas F, Taylor A, Binkowitz B and Ng J (1992) The effect of finasteride in men with benign prostatic hyperplasia. *N Engl J Med* **327**: 1185–1191

Gould VE, Doljanskaia V, Grooch G and Bostwick DG (1996) Immunolocalization of glycoprotein A-80 in prostatic carcinoma and prostatic intraepithelial neoplasia. *Hum Pathol* **27**: 547–552

Grino PB, Griffen JE and Wilson JD (1990) Testosterone at high concentrations interacts with the androgen receptor similarly to dihydrotestosterone. *Endocrinology* **126**: 1165–1172

Hall PA, Levision DA, Woods AL, Yu CC, Kellock DB, Watkins JA, Barnes DM, Gillett CE, Camplejohn R and Dover R (1990) Proliferating cell nuclear antigen (PCNA) immunolocalization in paraffin sections: an index of cell proliferation with evidence of deregulated expression in some neoplasms. *J Pathol* **162**: 285–294

Hovenian MS and Deming CL (1948) The heterologous growth of cancer of human prostate. *Surg Gynecol Obstet* **86**: 29–35

Huggins C and Hodges CV (1941) Studies on prostate cancer: effect of castration, of estrogen, and of androgen injection on serum phosphatases in metastatic carcinoma of the prostate. *Cancer Res* **1**: 293–297

Imperato-McGinley J, Guerrero L, Gautier T and Peterson RE (1974) 5-alpha reductase deficiency in man: an inherited form of male pseudohermaphroditism. *Science* **186**: 1213–1215

Keetch DW, Catalona WJ and Smith DS (1994) Serial prostatic biopsies in men with persistently elevated serum prostate specific antigen values. *J Urol* **151**: 1571–1574

Lamb JC, English H, Levandoski PL, Rhodes GR, Johnson RK and Isaacs JT (1992) Prostatic involution in rats induced by a novel 5-alpha reductase inhibitor, SK & F 105657: role for testosterone in the androgenic response. *Endocrinology* **130**: 685–694

Lepor IT, Williford WO, Barry M, Brawer MK, Dixon CM, Gormley G, Haakenson C, Machi M, Narayan P and Padley RJ (1996) The efficacy of terazosin, finasteride, or both in benign prostate hyperplasia. *N Engl J Med* **335**: 533–539

Lookingbill D, Demers L, Wang C, Leung A, Rittmaster RS and Santen RJ (1991) Clinical and biochemical parameters of androgen action in normal healthy Caucasian versus Chinese subjects. *J Clin Endocrinol Metab* **72**: 1242–1248

McNeal JE (1969) Origin and development of carcinoma in the prostate. *Cancer* **23**: 24–34

McNeal JE and Bostwick DG (1986) Intraductal dysplasia: a premalignant lesion of the prostate. *Hum Pathol* **17**: 64–71

Mehta CR and Patel NR (1983) A network algorithm for performing Fisher's exact test in RxC contingency tables. *J Am Statist Assoc* **78**: 427–434

Muir C, Waterhouse J, Mack T, Powell J and Whelan S (1987) *Cancer Incidence in Five Continents*, Vol. V. IARC: Lyon

Noble RL (1977) The development of prostatic adenocarcinoma in Nb rats following prolonged sex hormone administration. *Cancer Res* **37**: 1929–1933

Norman RW, Coakes KE, Wright AS and Rittmaster RS (1993) Androgen metabolism in men receiving finasteride before prostatectomy. *J Urol* **150**: 1736–1739

Pocock SJ and Simon R (1975) Sequential treatment assignment with balancing for prognostic factors in the controlled clinical trial. *Biometrics* **31**: 103–115

Presti JC, Fair WR, Androle G, Sogani PC, Seidmon EJ, Ferguson D, Ng J and Gormley GJ (1992) Multicenter, randomized, double-blind placebo controlled study to investigate the effect of finasteride (MK-906) on stage D prostate cancer. *J Urol* **148**: 1201–1204

Preston-Martin S, Pike MC, Ross RK, Jones PA and Henderson BE (1990) Increased cell division as a cause of human cancer. *Cancer Res* **50**: 7415–7421

Ross RK, McCurtis JW, Henderson BE, Menck HR, Mack TM and Martin SP (1979) Descriptive epidemiology of testicular and prostatic cancer in Los Angeles. *Br J Cancer* **39**: 284–292

Ross RK, Bernstein L, Judd H, Hanisch R, Pike MC and Henderson BE (1986) Serum T levels in healthy young black and white men. *J Natl Cancer Inst* **76**: 45–48

Ross RK, Bernstein L, Lobo RA, Shimizu H, Stanczyk F, Pike MC and Henderson BE (1992) 5-alpha reductase activity among Japanese and US white and black males. *Lancet* **339**: 887–890

Salem CE, Tomasic NA, Elmajian DA, Esrig D, Nichols PW, Taylor CR, Skinner DG, Roy-Burman P, Lieskovsky G and Cote RJ (1997) p53 protein and gene alterations in pathological stage C prostate carcinoma. *J Urol* **158**: 510–514

Shi S-R, Key ME and Kalra KL (1991) Antigen retrieval in formalin-fixed, paraffin-embedded tissues: an enhancement method for immunohistochemical staining based on microwave oven heating of tissue sections. *J Histochem Cytochem* **39**: 741–748

Stoner E and Finasteride Study Group (1994*a*) Three-year safety and efficacy data on the use of finasteride in the treatment of benign prostatic hyperplasia. *Urology* **43**: 284–294

Stoner E, Round E, Ferguson D and Gormley GJ (1994*b*) Clinical experience of the detection of prostate cancer in patients with benign prostatic hyperplasia treated with finasteride. *J Urol* **151**: 1296–1300

Taylor CR, Shi S-R, Chaiwun B, Young L, Imam SA and Cote RJ (1994) Strategies for improving the immunohistochemical staining of various intranuclear prognostic markers in formalin-paraffin sections: androgen receptor, estrogen receptor, progesterone receptor, p53 protein, proliferating cell nuclear antigen, and ki-67 antigen revealed by antigen retrieval techniques. *Hum Pathol* **25**: 263–270

Tempany CMC, Partin AW, Zerhouni EA, Zinreich SJ and Walsh PC (1993) The influence of finasteride on the volume of the peripheral and periurethral zones of the prostate in men with benign prostatic hyperplasia. *Prostate* **22**: 39–42

Tutrone RF, Ball RA, Ornitz DM, Leder P and Richie JP (1993) Benign prostate hyperplasia in a transgenic mouse: a new hormonally sensitive investigatory model. *J Urol* **149**: 633–639

Umekita Y, Hiipakka RA, Kokontis JM and Liao S (1996) Human prostate tumor growth in athymic mice: inhibition by androgens and stimulation by finasteride. *Proc Natl Acad Sci USA* **93**: 11802–11807

Walsh PC, Madden JD, Harrod MJ, Goldstein JL, MacDonald PC and Wilson JD (1974) Familial incomplete male pseudohermaphroditism, type 2: decreased dihydroT formation in pseudovaginal perineoscrotal hypospadias. *N Engl J Med* **291**: 944

Waseem NH and Lane DP (1990) Monoclonal antibody analysis of the proliferating cell nuclear antigen (PCNA). *J Cell Sci* **96**: 121–129

Wilson JD (1989) Sexual differentiation of the gonads and the reproductive tract. *Biol Neonate* **55**: 322–330

© Cancer Research Campaign 1998